# Translation and Validation of the Health Literacy Score-14 Questionnaire for Vietnamese Patients with Diabetes

**DOI:** 10.31662/jmaj.2023-0148

**Published:** 2024-04-10

**Authors:** Khoa Tuan Vo, Khue Thy Nguyen, Hirohide Yokokawa, Aya Goto, Toshio Naito

**Affiliations:** 1 Department of Endocrinology, People’s Hospital 115, Ho Chi Minh City, Vietnam; 2 Ho Chi Minh City Medical Association, Ho Chi Minh City, Vietnam; 3 Juntendo University School of Medicine, Tokyo, Japan; 4 Fukushima Medical University Center for Integrated Science and Humanities, Fukushima, Japan

**Keywords:** health literacy, diabetes, questionnaire design, validity, Vietnam

## Abstract

**Introduction:**

Health literacy (HL) is a crucial indicator for health promotion and diabetes care improvement, but the available measurements are mostly in English. This study aimed to translate and validate the 14-item Health Literacy Scale (HLS-14) questionnaire from English to Vietnamese for patients with diabetes in Vietnam.

**Methods:**

We translated HLS-14 into Vietnamese in accordance with the World Health Organization guidelines and conducted a cross-sectional survey among 571 outpatients with type 2 diabetes using the HLS-14 Vietnamese version (HLS-14 VN). The reliability and validity of the tool were assessed using Cronbach’s alpha, composite reliability (CR), average variance extracted (AVE), and maximum shared variance (MSV), and confirmatory analysis was conducted.

**Results:**

Cronbach’s alpha coefficients for the three subscales as in the original version were 0.931, 0.810, and 0.928 for functional HL, communicative HL, and critical HL, respectively. However, AVE for critical HL was 0.488, which improved to 0.516 after the removal of one item in the communicative HL. For all subscales in the revised 13-item version (HLS-13 VN), CR was above 0.8, AVE was above 0.5, and MSV was less than AVE. Confirmatory analysis of HLS-13 VN revealed an acceptable fit with comparative fit index of 0.983, goodness-of-fit index of 0.963, and root mean squared error of approximation of 0.058.

**Conclusions:**

The reliability and validity of HLS-13 VN were confirmed. The tool is suitable for use in clinical settings in Vietnam to assess multidimensional HL in patients with type 2 diabetes.

## Introduction

Having an adequate level of ability to understand health information motivates people to better understand their own health and empowers them in self-management ^(1), (2)^. Thus, individuals are recommended to have an adequate level of heath literacy (HL), which is defined by the World Health Organization as “the cognitive and social skills which determine the motivation and ability of individuals to gain access to, understand and use information in ways which promote and maintain good health” ^(3)^. It has long been known that inadequate HL exerts negative effects on health outcomes and healthcare service utility ^(4)^.

In the modern age, HL requires not only skills in reading, writing, and numeracy but also an ability to use the Internet ^(1)^. Various tools to screen the HL levels of people and patients have been designed and used ^(5)^. For short- and long-term diabetes control, although routine screening is not recommended, clinicians should be aware of both the HL level of their patients and the importance of their own HL skills to convey health information in an accessible manner. HL promotion requires bilateral communication between service providers and patients so as to provide individualized services to users. In addition, familiarity with HL is recommended not only for clinicians at health and medical institutes but also for all public health professionals to provide a driving force for health promotion and support community health and policymakers to reinforce HL promotion at the organizational and policy levels ^(6), (7)^.

Nutbeam developed an HL model including three domains: functional HL for examining reading and writing skills, communicative HL for extracting and communicating information, and critical HL for analyzing and making decisions ^(8)^. Using Nutbeam’s conceptual framework as a basis, Ishikawa et al. devised an instrument called the 14-item Health Literacy Scale (HLS-14) to be applied among patients with diabetes ^(9)^. HLS-14 was then revised and validated for use among the general public in Japan by Suka et al. ^(10)^.

In Vietnam, HL is considered a new approach to respond to a rapid increase in noncommunicable diseases, particularly diabetes mellitus ^(11)^. As a chronic progressive disease, it requires patients to perform self-management in their daily activities. Their HL skills can be the foundation for both treatment adherence and self-efficacy in disease management ^(2), (12), (13)^. Recently, the comprehensive short-form HL survey tool (HLS-SF12) was developed from the 47-item European Health Literacy Questionnaire (HLS-EU Q47) and validated in the Vietnamese general population ^(14)^. However, the HLS-SF12 has not been applied in clinical settings targeting patient groups, such as those with diabetes. Therefore, this study aimed to translate and validate the HLS-14, which has been validated for Japanese patients with diabetes, for use among Vietnamese patients with diabetes.

## Materials and Methods

### Translation process

The original version of the questionnaire was translated into Japanese, and its English version was published ^(9)^. We followed the translation processes proposed in the World Health Organization (WHO)’s 2016 guidelines ^(15)^. Two translators independently translated the questionnaire from English into Vietnamese, then two principal investigators compared and discussed criteria such as clarity, common language use, and conceptual equivalence to create a draft Vietnamese version. A total of 10 volunteer patients with diabetes evaluated the draft to identify words and questions that were difficult to understand or answer and made suggestions for improvements. Some minor changes were made to develop the preliminary Vietnamese version, which was backtranslated into English by two health professionals working independently. Subsequently, we reviewed and discussed the differences between the translations and the original content, and the finalized Vietnamese version (HLS-14 VN) was approved by all research team members before being subjected to a validity study.

### Study setting and participants

This was a hospital-based cross-sectional study conducted at the outpatient department of People’s Hospital 115, Ho Chi Minh City, Vietnam, between June 2020 and February 2021. People’s Hospital 115 is one of the largest tertiary general hospitals in Ho Chi Minh City, with 1,600 beds. Participants were selected from patients who fulfilled the inclusion criteria: (1) having type 2 diabetes and (2) aged 18 years or above. Patients who had difficulty communicating in the Vietnamese language as a result of cognitive, hearing, and/or visual impairment were excluded.

Participants were interviewed by health professionals who had been informed of the study aims and methods. Demographic data, clinical parameters, and items related to diabetes were collected using a standardized self-administered questionnaire. The HLS-14 VN contained five items each for functional and communicative HL and four for critical HL. A five-point Likert scale was employed for the answer options for each item: strongly disagree, disagree, not sure, agree, and strongly agree. The scores for the items in each subscale were summed to obtain subscale totals.

### Data analysis

The reliability of the HLS-14 VN was evaluated using Cronbach’s alpha coefficient ^(16)^, corrected item-total (IT) correlation, and Cronbach’s alpha if item was deleted (i.e., reevaluated Cronbach’s alpha after scale items are individually removed). The IT correlation indicates the strength of the association between each item and the summated score for all other items. Items with an IT correlation below 0.30 are considered not to be satisfactorily correlated with the entire scale, suggesting the need to remove them ^(17)^. Items for which Cronbach’s alpha increases if the item is deleted from the scale are also removed.

Exploratory factor analysis (EFA) was conducted to establish the factor structure. To determine whether the measures are suitable for factor analysis, the Kaiser-Meyer-Olkin (KMO) test was employed to measure sampling adequacy, and Bartlett’s test of sphericity was used to assess the factorability of the data. A KMO value close to 1 and a significant statistical result for Bartlett’s sphericity test indicate suitability for factor analysis. Principle component analysis with promax rotation was conducted to establish the factor structure of the instrument. Eigenvalues were determined for each factor, representing the total amount of variance explained by each factor, with the total amount of variability equal to the number of original variables in the analysis. The Kaiser rule was adopted, which states that only those factors with eigenvalues greater than 1 should be retained. Furthermore, a general rule was applied to retain the factors that contribute at least 70% of the total variance.

Construct validity was assessed using confirmatory factor analysis (CFA) based on the original three-factor model ^(9)^. The following indices were used: ratio of chi-squared minimum and degree of freedom (CMIN/DF), goodness-of-fit index (GFI), comparative fit index (CFI), Tucker-Lewis’s coefficient index (TLI), root mean squared error of approximation (RMSEA), and probability of close fit (PCLOSE). The following values indicated a good fit: CMIN/DF < 3; GFI, CFI, and TLI > 0.90; RMSEA < 0.08; and PCLOSE > 0.05 ^(18)^. The modification indices were calculated to improve model fit by allowing correlations between within-factor error terms.

Convergent validity assesses the extent to which multiple measures of a construct are associated. In this study, convergent validity of the factors was assessed using the measures of factor loading (FL) of items, composite reliability (CR), and average variance extracted (AVE) ^(19)^. CR value ≥ 0.70 and AVE value ≥ 0.50 were considered satisfactory whereas FL value > 0.5 was acceptable ^(20)^. Discriminant validity refers to the degree to which a measure does not correlate with another measure whose underlying construct is theoretically unrelated to it. To evaluate discriminant validity, the square root of AVE of each factor was compared with the correlation coefficient between factors (ρ_ij_) as well as AVE and maximum shared variance (MSV). Discriminant validity was established when AVE < MSV and the square root of AVE > all of (ρ_ij_) in the same row and column ^(19)^.

All analyses were conducted using IBM SPSS version 16.0, whereas CFA analysis was conducted using IBM SPSS Amos version 20.0.

### Ethical considerations

The study was reviewed and approved by the Scientific and Ethical Committee of People’s Hospital 115 (Number 1903/QD-BV115 dated 27/09/2019). Written informed consent was obtained from all participants.

## Results

Following the translation process, there were no substantive changes in the content of the HLS-14 VN compared with the original version. However, in some instances, wording was revised to improve ease of understanding in Vietnamese. For example, the verb “read” from items fhl1, fhl4, and fhl5 could be confused with the meaning “read and understand”; therefore, this was elucidated in the HLS-14 VN. For the functional HL scale, volunteer patients suggested the inclusion of instruction cards or leaflets as examples of literature that needs to be understood. Some minor formatting adjustments were also made to improve the questionnaire layout.

Of the 575 eligible subjects, 571 completed the questionnaire (response rate: 99.3%). As presented in Table 1, the mean age of the respondents was 59 years, and 275 (48.2%) of them were men. The proportions of those who did not smoke and drink were as high as 79.1% and 72.2%, respectively, but the proportion of those doing regular physical activities was only 16.3%. In terms of disease status, the mean diabetes duration was 5.5 years and the mean HbA1c was 8.6%.

**Table 1.** Characteristics of Participants (n = 571).

Variables	*Mean ± SD* or n (%)
Age (years)	*59.0 ± 13.8*
Sex	
Male	275 (48.2)
Occupation	
Blue collar	214 (37.6)
White collar	140 (24.6)
Retired	215 (37.8)
Educational level	
Primary	79 (13.9)
Junior high school	125 (22.0)
Secondary high school	160 (28.1)
University	205 (36.0)
Smoking status	
Yes	40 (7.0)
No	450 (79.1)
Ex	79 (13.9)
Alcohol consumption	
Regularly	17 (3.0)
Sometimes	141 (24.8)
No	411 (72.2)
Physical activities	
Active	93 (16.3)
Low active	260 (45.7)
Sedentary	216 (38.0)
Diabetes duration (years)	*5.5 ± 4.9*
Body mass index (kg/m^2^)	*26.3 ± 3.3*
Blood glucose (mmol/L)	*7.6 ± 2.0*
Hemoglobin A1c (%)	*8.6 ± 3.9*

SD: standard deviation

The mean scores of functional, communicative, and critical HL were 3.57, 3.99, and 4.33, and Cronbach’s alpha values were 0.931, 0.810, and 0.928, respectively (Table 2). Cronbach’s alpha if item deleted only increased in value when the first item of communicative HL (chl1) was omitted; this item also showed the lowest IT correlation. Notably, the KMO index was 0.85 and Bartlett’s test of sphericity was significant (n = 571, χ^2^ = 5827, df = 91; p < 0.0001). After extraction and rotation, three components in the dataset had eigenvalues greater than 1. The first component explained 39.89% of the total variance, with an eigenvalue of 5.58. The second component explained 63.41% variance, with an eigenvalue of 3.29. Furthermore, the third component explained 9.48% variance, with an eigenvalue of 1.33. The overall result exhibited 72.88% of the total estimated variance with the three domains. Table 3 demonstrates that the FL of all items was within the range of 0.711-0.960.

**Table 2.** Distributional Properties and Internal Consistency.

Subscales and items	Mean ± SD	Item-total correlation	Cronbach’s alpha if item deleted	Cronbach’s alpha
FHL (What you thought when reading instructions or leaflets from hospitals/pharmacies)	3.57 ± 0.97			0.931
fhl1 (Print was too small to read)	3.50 ± 1.13	0.818	0.915	
fhl2 (There were characters and words that you did not know)	3.63 ± 1.08	0.834	0.912	
fhl3 (Content was too difficult)	3.68 ± 1.00	0.814	0.917	
fhl4 (It took a long time to read and understand)	3.41 ± 1.66	0.806	0.918	
fhl5 (Needed someone to help to read)	3.62 ± 1.10	0.824	0.914	
CHL (What was done since diagnosed with diabetes)	3.99 ± 0.59			0.810
chl1 (Collected information from various sources)	3.68 ± 1.00	0.534	0.811	
chl2 (Extracted the information you wanted)	3.98 ± 0.72	0.596	0.774	
chl3 (Understood the obtained information)	4.08 ± 0.71	0.673	0.753	
chl4 (Communicated opinions about your illness)	4.16 ± 0.68	0.628	0.767	
chl5 (Applied the obtained information to your daily life)	4.06 ± 0.74	0.624	0.766	
CRHL (What you thought about the information obtained since diagnosed with diabetes)	4.33 ± 0.64			0.928
crhl1 (Whether the information was applicable)	4.26 ± 0.70	0.797	0.917	
crhl2 (Whether the information was credible)	4.39 ± 0.73	0.824	0.908	
crhl3 (Whether the information was valid and reliable)	4.37 ± 0.69	0.890	0.887	
crhl4 (Sought information for making health-related decisions)	4.38 ± 0.70	0.816	0.911	

fhl: functioning health literacy, chl: communicative health literacy, crhl: critical health literacy, SD: standard deviation

**Table 3.** Factor Loadings after Promax Rotation with Kaiser Normalization.

Items	Factor loading		
	Factor 1	Factor 2	Factor 3
fhl1	0.897		
fhl2	0.906		
fhl3	0.873		
fhl4	0.878		
fhl5	0.887		
chl1			0.827
chl2			0.739
chl3			0.711
chl4			0.730
chl5			0.755
crhl1		0.838	
crhl2		0.891	
crhl3		0.960	
crhl4		0.931	

fhl: functioning health literacy, chl: communicative health literacy, crhl: critical health literacy

**Figure 1.**
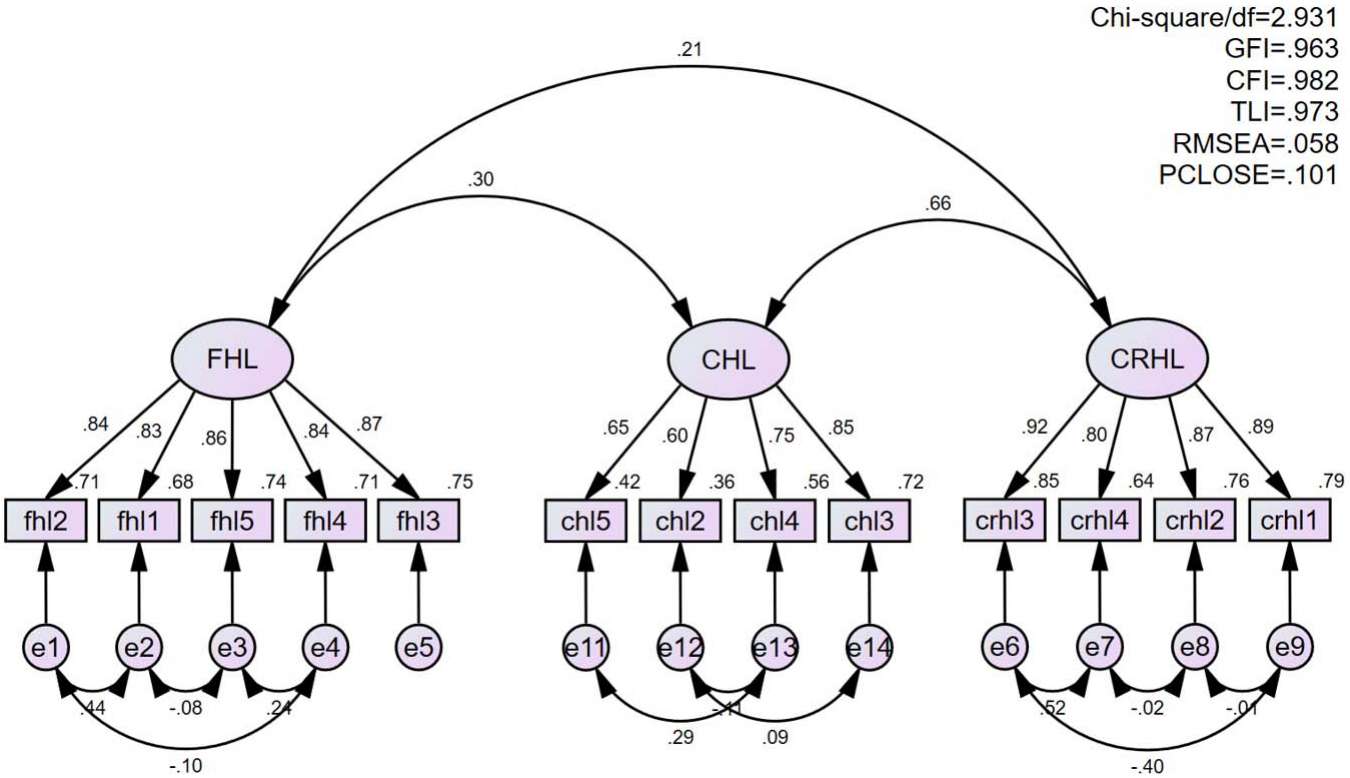
Path diagram of the confirmatory factor model for the 13-item version (HLS-13 VN) Rectangles indicate the observed variables (items) and ellipses the latent constructs (factors). Values on the single-head arrows leading from the factors to the items are standardized FLs. Values on the curved double-headed arrows are correlations between factors/errors. fhl: functioning health literacy, chl: communicative health literacy, crhl: critical health literacy, df: degree of freedom, GFI: goodness-of-fit index, CFI: comparative fit index, TLI: Tucker-Lewis index, RMSEA: root mean squared error of approximation, PCLOSE: probability of close fit.

The indicators in Table 4 suggest that Ishikawa’s original 14-item version ^(9)^ (Model 1) did not satisfy Hu et al.’s criteria for good fit ^(18)^. The AVE for critical HL in the 14-item version was 0.488, indicating that the convergent validity for the subscale was unsatisfactory (Table 5). After checking the standardized regression weight, the first item of communicative HL was discarded with the lowest value of 0.533. The modified 13-item version (Model 2) satisfied Hu et al.’s criteria (Table 4; GFI = 0.963, CFI = 0.983, and RMSEA = 0.058), and for all subscales, CR was above 0.8, AVE was above 0.5, and MSV was less than AVE (Table 5). In addition, recalculated Cronbach’s alpha coefficients were 0.93, 0.81, and 0.94 for functional, communicative, and critical HL, respectively. Figure 1 presents the path diagram of the final 13-item version (HLS-13 VN).

**Table 4.** Model Fit Indices in Confirmatory Factor Analysis.

Indicators	Criteria of Hu et al. ^(18)^	Model 1 Original model (3 factors, 14 items)	Model 2 Proposed model (3 factors, 13 items)
Chi-squared minimum/degree of freedom (CMIN/DF)	<3.00	7.730	2.931
Goodness-of-fit index (GFI)	>0.90	0.877	0.963
Comparative fit index (CFI)	>0.90	0.914	0.983
Tucker-Lewis index (TLI)	>0.90	0.894	0.973
Root mean squared error of approximation (RMSEA)	<0.08	0.109	0.058
Probability of close fit (PCLOSE)	>0.05	0.000	0.101

**Table 5.** Convergent and Discriminant Validity.

				Square root of AVE	Correlation		
Subscales	CR	AVE	MSV		fhl	chl	crhl
Original model (3 factors, 14 items)							
fhl	0.932	0.733	0.073	0.856	0.856		
chl	0.823	0.488	0.410	0.698	0.271	0.698	
crhl	0.928	0.765	0.410	0.875	0.196	0.640	0.875
Proposed model (3 factors, 13 items)							
fhl	0.927	0.717	0.090	0.847	0.847		
chl	0.807	0.516	0.437	0.718	0.299	0.718	
crhl	0.926	0.759	0.437	0.871	0.208	0.661	0.871

fhl: functioning health literacy, chl: communicative health literacy, crhl: critical health literacy, CR: composite reliability, AVE: average variance extracted, MSV: maximum shared variance

## Discussion

The newly developed HLS-13 VN is a unique tool for assessing the three dimensions of HL level among Vietnamese patients with diabetes and can be used to promote communication between diabetes care providers and patients in the Vietnamese context. The translation process was performed in accordance with the WHO translation protocol, and some changes were made in the translated version to reflect differences in culture and language (e.g., the word “read” in items fhl1, fhl4, and fhl5). Furthermore, a small group of volunteer patients was interviewed about difficult-to-understand words, phrases, or questions to make further minor modifications. Carefully discussing the translation in this manner could help improve the quality of a local version of a survey instrument ^(21)^. Regarding the survey method, we received important feedback from patients about avoiding morning as the time for questionnaire completion (many outpatients indicated that it was difficult for them to concentrate on the survey as a result of undertaking routine fasting for their morning visit to the hospital) and that it would be helpful to receive a handout explaining how to correctly fill in the survey. This feedback shows that it is important to interactively communicate with patients from the survey planning and preparation stages.

In terms of EFA, KMO value of over 0.8 and a significant Bartlett’s test result indicated that our dataset was adequate for factor analysis ^(22)^. Eigenvalues greater than 1 for three factors represented the amount of total variance in each item that can be explained by the principal component. Furthermore, these three factors accounted for most of the total variability in the dataset. By then applying CFA to Ishikawa et al.’s original HLS-14 ^(9)^, it was found that five (CMIN/DF, GFI, TLI, RMSEA, and PCLOSE) out of six fit indices (except CFI) indicated a poor fit. Such differences between the original and translated versions of HL measurement were reported in a previous study ^(23)^. To improve the model fit, the first communicative HL item (chl1: “Collected information from various sources”) was deleted to increase all fit index values to acceptable levels. A similar modification was reported for the HLS-14 Brazilian-Portuguese version ^(24)^. Diabetes-related information comes from diverse sources, including physicians, healthcare professionals, peer patients, diabetes education programs, relatives and friends, mass media, the Internet, leaflets, and newsletters. At present, many patients with diabetes can easily find health information in the Internet. Vietnam is also a country where the Internet is widely used; the number of users in 2021 was reported to be 69 million out of the total population of 96 million ^(25)^. With digital information becoming the single most common information source, a survey item asking regarding the collection of information from various sources may no longer be necessary.

In addition to the aforementioned good model fit, our revised HLS-13 VN exhibited satisfactory convergent and discriminant validity. The four indices (CR, MSV, AVE, and square root of AVE) indicated that each subscale was unidimensional and did not have overlap items and that Cronbach’s alpha coefficient exhibited good internal consistency for all three subscales. A similar finding was obtained in the development of Iranian ^(26)^, Dutch ^(27)^, and Brazilian-Portuguese ^(24)^ versions. Our study adds to the existing evidence that for translated versions of the scale, the modified HLS-13 is suitable for HL measurement among patients. People with diabetes need to have the ability to continue self-learning, self-care, and self-management after being provided with oral and written instructions by health professionals ^(28)^. The HLS-13 VN lists the practical skills needed to use health information in daily life. We recommend wider usage of the tool at health and medical institutes to make healthcare providers more aware of their clients’ HL capacity and better able to meet their needs. It would also help patients become aware of the importance of managing their own health.

This study has several methodological limitations. First, the validation sample consisted of outpatients with diabetes at a single hospital during the COVID-19 outbreak. The generalizability of the present findings is therefore limited. Second, those with inadequate Vietnamese language skills are often the most vulnerable portion of the population, requiring special attention and additional care in terms of health promotion. Exclusion of this group from the target population could affect the study implications. A wider and repeated application and evaluation of the devised instrument are warranted ^(29)^.

### Practical implications

The HLS-14 is used to measure different domains of HL. For Vietnamese healthcare professionals, the shorter version of the HLS-13 VN is easy to apply. The questionnaire can be widely disseminated by using digital tools such as a QR code linked to an Internet-based survey. If healthcare providers can develop an understanding of the HL level of their patients with diabetes, this allows them to improve services and communication. For decision-makers from organizations and (local) governments, this instrument can play a beneficial role in monitoring health promotion programs and optimizing diabetes care strategies.

### Conclusion

To the best of our knowledge, this is the first study on a multidimensional HL assessment tool adapted for Vietnamese patients with diabetes. The HLS-13 VN exhibited sufficient reliability and validity. The tool is suitable for use in clinical settings to raise awareness among healthcare professionals of the HL needs of their clients, enabling them to improve and individualize care. Further research is warranted among more diverse populations.

## Article Information

### Conflicts of Interest

None

### Acknowledgement

This study was conducted as a part of the Japan International Cooperation Agency’s Partnership Program (Local Government Type) “Capacity building toward evidence-based medicine among health care professionals at University of Medicine and Pharmacy, Ho Chi Minh City and its related institutes” (PI: AG) and a 2020 Grant-in-Aid for Scientific Research (C) (PI: HY, No. 20K10539). We thank Oliver Stanyon for editing the draft of this manuscript.

### Author Contributions

KTV planned the study, collected and analyzed data, and wrote the manuscript. HY, KTN, GA, and TN offered advice for study planning and data analysis and contributed to the finalization of the manuscript. All authors have read and approved the final draft of the manuscript.

### Approval by Institutional Review Board (IRB)

This study was reviewed and approved by the Scientific and Ethical Committee of People’s Hospital 115 (Number 1903/QD-BV115 dated 27/09/2019).

### Disclaimer

Aya Goto is one of the Editors of JMA Journal and on the journal’s Editorial Staff. She was not involved in the editorial evaluation or decision to accept this article for publication at all.
