# Erratum: 2024;7(2):242-9 (DOI: 10.31662/jmaj.2023-0148)

**DOI:** 10.31662/jmaj.e0004

**Published:** 2026-05-29

**Authors:** Khoa Tuan Vo, Khue Thy Nguyen, Hirohide Yokokawa, Aya Goto, Toshio Naito

**Affiliations:** 1 Department of Endocrinology, People’s Hospital 115, Ho Chi Minh City, Vietnam; 2 Juntendo University School of Medicine, Tokyo, Japan; 3 Ho Chi Minh City Medical Association, Ho Chi Minh City, Vietnam; 4 Fukushima Medical University Center for Integrated Science and Humanities, Fukushima, Japan

Article: Tuan Vo K, Thy Nguyen K, Yokokawa H, et al. Translation and validation of the health literacy score-14 questionnaire for Vietnamese patients with diabetes. JMA J. 2024;7(2):242-9.

Correction 1: Author affiliations.

Original:

Khoa Tuan Vo^1^, Khue Thy Nguyen^2^, Hirohide Yokokawa^3^, Aya Goto^4^, and Toshio Naito^3^

^1^Department of Endocrinology, People’s Hospital 115, Ho Chi Minh City, Vietnam

^2^Ho Chi Minh City Medical Association, Ho Chi Minh City, Vietnam

^3^Juntendo University School of Medicine, Tokyo, Japan

^4^Fukushima Medical University Center for Integrated Science and Humanities, Fukushima, Japan

Corrected:

Khoa Tuan Vo^1,2^, Khue Thy Nguyen^3^, Hirohide Yokokawa^2^, Aya Goto^4^, and Toshio Naito^2^

^1^Department of Endocrinology, People’s Hospital 115, Ho Chi Minh City, Vietnam

^2^Juntendo University School of Medicine, Tokyo, Japan

^3^Ho Chi Minh City Medical Association, Ho Chi Minh City, Vietnam

^4^Fukushima Medical University Center for Integrated Science and Humanities, Fukushima, Japan

